# Whole‐Brain Fractional Anisotropy, Centered on the Superior Longitudinal Fasciculus, Is Positively Correlated With Urban Aesthetic Perception

**DOI:** 10.1002/brb3.71446

**Published:** 2026-04-27

**Authors:** Keisuke Kokubun, Kiyotaka Nemoto, Maya Okamoto, Yoshinori Yamakawa

**Affiliations:** ^1^ Graduate School of Management Kyoto University Kyoto Japan; ^2^ Department of Medical Informatics and Management and Psychiatry, Institute of Medicine University of Tsukuba Tsukuba Japan; ^3^ Nocturne Capital Tsukuba Japan; ^4^ Institute of Innovative Research Institute of Science Tokyo Meguro, Tokyo Japan; ^5^ ImPACT Program of Council for Science Technology and Innovation (Cabinet Office, Government of Japan) Chiyoda, Tokyo Japan; ^6^ Office for Academic and Industrial Innovation Kobe University Kobe Japan; ^7^ Brain Impact Kyoto Japan

**Keywords:** diffusion tensor imaging, fractional anisotropy, magnetic resonance imaging, superior longitudinal fasciculus, urban aesthetics

## Abstract

**Introduction:**

With the growth of the global urban population, interest in urban aesthetics has been steadily increasing. Previous studies using functional magnetic resonance imaging (fMRI) to examine brain responses have suggested that various brain regions may be involved in the perception of urban aesthetics. However, the relationship between brain structure and urban aesthetics remains largely unexplored.

**Methods:**

In this study, we hypothesized that fractional anisotropy (FA) in the whole brain and in the superior longitudinal fasciculus (SLF), reflecting large‐scale structural connectivity across brain regions, would be associated with subjective evaluations of urban aesthetics. To test this hypothesis, we conducted diffusion tensor imaging (DTI) using magnetic resonance imaging (MRI) in a sample of 82 healthy adults recruited in Japan.

**Results:**

The results showed that both whole‐brain FA and SLF FA were positively correlated with the Aesthetic Quality Scale, which reflects urban aesthetics.

**Conclusion:**

This study is the first to demonstrate that urban aesthetics may be associated with white matter microstructure.

## Graphical abstract

This study examines the association between urban aesthetic perception and white matter microstructure in healthy adults. Diffusion tensor imaging reveals that both whole‐brain fractional anisotropy and superior longitudinal fasciculus integrity are positively related to subjective evaluations of urban aesthetic quality, suggesting that everyday environmental experiences may be reflected in structural brain connectivity.

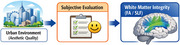

## 1 Introduction

The global urban population has been increasing year by year, rising from approximately 4 billion in 2015 to an estimated 5 billion in 2030 and 6 billion by 2040 (United Nations 2018). As a result, public interest in urban aesthetics has been steadily growing. The idea that the beauty of cities and architecture profoundly influences human emotions and behavior has long been recognized (Cooper and Burton 2014). Although beautiful urban spaces can enhance comfort and well‐being, disordered landscapes and stressful urban environments may have adverse effects on mental health. With the rise of cognitive neuroscience approaches in recent years, empirical studies have begun to accumulate on how aesthetic experiences of cities and architecture are related to brain structure and function. In the present study, aesthetic perception was operationalized as subjective evaluations of neighborhood aesthetic quality measured using the aesthetic quality subscale of the Neighborhood Environment Scale. Although aesthetic perception may include broader domains such as artistic appreciation, architectural aesthetics, and landscape perception, the present study focuses specifically on perceived aesthetic quality of everyday urban environments.

First, when considering urban aesthetics from the perspective of landscape, it is essential to take into account visual‐related regions. Visual processing is integrated into higher level regions such as the parahippocampal cortex, retrosplenial cortex, and occipital regions (Marchette et al. 2015). Among these, the parahippocampal cortex responds selectively to environmental scenes such as landscapes, building interiors, and urban spaces and plays an important role in spatial navigation (Mégevand et al. 2014). The occipital regions are involved in processing perceptual features such as building materials, windows, and architectural motifs, which may contribute to the recognition of building interiors and exteriors, and they respond more strongly to edges or areas with high visual contrast than to regions of uniform luminance within a scene (Geisler 2007). The retrosplenial cortex, in turn, acquires information that enables people to orient themselves within remembered or imagined spatial environments (Marchette et al. 2015).

However, vision alone is not sufficient to evaluate urban beauty. The emotions people experience in the presence of beautiful architecture may be mediated by the brain's reward circuitry. In a meta‐analysis of neuroimaging studies investigating positive aesthetic evaluation, Brown et al. (2011) argued that the processing of aesthetic emotions may occur through a core neural circuit that includes the orbitofrontal cortex (OFC), anterior cingulate cortex (ACC), and anterior insula. Subsequent functional magnetic resonance imaging (fMRI) studies have shown that curved building interiors are more likely to be judged as beautiful and pleasant than rectilinear spaces, and that the aesthetic evaluation of curved rooms is associated with increased activation in the ACC along with visual areas such as the lingual gyrus and calcarine gyrus (Vartanian et al. 2013). Furthermore, open rooms were found to activate temporal lobe structures related to visual processing, such as the left middle temporal gyrus and right superior temporal gyrus (STG), whereas high ceilings activated structures associated with visuospatial attention and exploration, including the left precuneus and left middle frontal gyrus (Vartanian et al. 2015).

In addition, the study by Vartanian et al. demonstrated that judgments of architectural beauty are influenced by activity in brain regions such as the prefrontal cortex (PFC), including the frontopolar cortex and superior frontal gyrus, as well as regions involved in memory retrieval such as the parahippocampal cortex (Vartanian et al. 2013). Activation of the PFC suggests that conscious reasoning and analysis may play an important role in aesthetic judgment, whereas activation of the parahippocampal cortex implies that memories generated from education and past experience may influence this analytic process. Increased activity in both of these neural regions indicates that aesthetic judgments are particularly shaped by inputs from knowledge‐based semantic systems, such as expertise, cultural tendencies, and an understanding of the intended function of a building (Vartanian et al. 2013).

With regard to aesthetic experiences such as art, previous studies have reported that not only the medial orbitofrontal cortex (mOFC) or ventromedial prefrontal cortex (vmPFC), which are activated during both musical and visual aesthetic appreciation (Ishizu and Zeki 2011; Kawabata and Zeki 2004), but also other brain regions, including the precuneus, ACC, and temporoparietal junction (TPJ), are involved in the aesthetic appreciation of artworks (Silveira et al. 2015). An earlier meta‐analysis identified a network of regions spanning from the occipital to the frontal lobes, suggesting that aesthetic experience is not confined to specific reward‐related regions but rather engages a broad range of brain regions (Boccia et al. 2016). A more recent meta‐analysis showed that the frontal lobe is more active during visual art appreciation, whereas the striatal regions and STG are more active during music appreciation. Furthermore, it identified the frontal lobe, posterior cingulate cortex (PCC), inferior occipital gyrus (IOG)/inferior frontal gyrus (IFG), hippocampus, and caudate nucleus as key hubs in visually induced aesthetic experiences, forming a limbic–frontal–striatal evaluative network (Andrews‐Hanna et al. 2010; Lebreton et al. 2009).

Thus, previous studies have demonstrated that a wide range of brain regions are involved in both urban aesthetics and aesthetic experiences. However, these findings are mainly derived from fMRI studies that measure brain responses, and very few prior studies have examined the relationship between brain structure and either urban aesthetics or aesthetic experiences using magnetic resonance imaging (MRI). An exception is Kokubun et al. (2025), which showed that gray matter volume (GMV) in the putamen and inferior temporal gyrus was correlated with subjective aesthetic appreciation. Interestingly, these regions largely overlapped with those identified in an earlier fMRI study showing that viewing images reflecting subjective “sublimity,” rather than beauty, activated the inferior temporal cortex, posterior hippocampus, and other subcortical regions, as well as the caudate nucleus and putamen (Ishizu and Zeki 2014). This finding may relate to earlier claims that aesthetic judgment can be influenced by expertise and cultural tendencies (Vartanian et al. 2013). In other words, beauty and sublimity may be perceived as either distant or close depending on an individual's knowledge and preferences, suggesting that such participant effects could affect the reproducibility of results.

Conversely, in order to predict the relationship between beauty—such as urban aesthetics, which involves multiple sensory elements including vision, audition, and olfaction—and brain structure, it may be justified to adopt a strategy that designates neural pathways connecting multiple regions as regions of interest, taking into account that individual differences in aesthetic perception could further amplify this diversity. Alternatively, given that fMRI studies have revealed the widespread involvement of the brain in aesthetic experiences, it may also be justified to focus on the whole brain rather than on specific regions or fiber tracts. However, quantified whole‐brain GMV merely reflects the average size of GMV across individual regions and does not capture the quality of inter‐regional networks. In contrast, indices that reflect white matter (WM) microstructure, such as fractional anisotropy (FA) obtained through diffusion tensor imaging (DTI), capture the integrity of neural pathways essential for cognition and emotional regulation (Alnæs et al. 2018; Jiang et al. 2022). Low FA is generally regarded as a marker of tract disruption and demyelination and has been linked to declines in cognitive ability (Carozza et al. 2025; Schmithorst and Yuan 2010) as well as increased risk of psychiatric disorders (Pasi et al. 2016; White et al. 2008; Zhang et al. 2018). Pineda et al. (2021) further reported that although housing quality was associated with WM integrity, it was not related to GMV and argued that alterations in WM integrity in healthy adults may serve as a sensitive biomarker that precedes GMV loss.

In addition, research on green space exposure has utilized DTI‐derived measures such as FA, suggesting potential applicability to studies of urban aesthetics. However, in the context of green space exposure, DTI measures have mainly been emphasized in relation to stress rather than beauty. The beneficial effects of green space exposure are explained by Stress Reduction Theory (SRT) (Nieuwenhuijsen et al. 2017). SRT describes how exposure to nature induces changes in emotional states and physiological activity levels, thereby facilitating stress recovery (Ulrich et al. 1991). The stress‐reduction processes described by SRT may involve structural strengthening of stress‐sensitive neural circuits, including the limbic system and sensorimotor integration pathways (Kühn et al. 2017; Lederbogen et al. 2011). Accordingly, SRT suggests that exposure to urban green spaces plays a key role in eliciting rapid affective and emotional responses and initiating recovery from mental fatigue and stress (Ulrich et al. 1991). More recent studies have further shown that exposure to urban green spaces may promote WM integrity, emphasizing the possibility that stress reduction provided by green space exposure contributes to improvements in WM microstructure (Liu et al. 2025).

Urban aesthetics may carry different policy implications depending on whether they are perceived as positive or negative. A recent meta‐analysis examining negative aesthetic evaluations or the presentation and evaluation of negative non‐art images revealed activation not only in the right fusiform gyrus but also across frontal, occipital, temporal, and subcortical regions (Slaby et al. 2025). In this context, urban aesthetics may involve negative elements such as air pollution and noise, which must be taken into consideration. Previous studies have reported associations between air pollution and alterations in gray and WM structure (Cserbik et al. 2020; Guxens et al. 2018; Lubczyńska et al. 2021), including WM microstructure (Binter et al. 2024; Burnor et al. 2021; Lubczyńska et al. 2020). More recent findings have shown that exposure to noise during pregnancy and childhood reduces FA before adolescence (Binter et al. 2024). Noise exposure increases the release of stress hormones (Hahad et al. 2019) and may affect fetal brain development, particularly the microstructural connectivity between limbic and frontotemporal networks (Lautarescu et al. 2020). Although most of these studies have focused on participants during pregnancy or childhood, Kokubun, Nemoto, and Yamakawa (2025) recently demonstrated that subjective local environmental factors, including pollution and noise, were positively correlated with whole‐brain and cerebellar GMV, as well as FA in the posterior thalamic radiation and sagittal stratum in healthy adults.

As summarized above, FA—an index reflecting the integrity of neural pathways (Alnæs et al. 2018; Jiang et al. 2022), positively associated with urban green space exposure (Binter et al. 2024; Liu et al. 2025), negatively associated with exposure to air pollution (Binter et al. 2024) and noise (Lautarescu et al. 2020), and considered a potential biomarker that precedes GMV loss (Pineda et al. 2021)—holds strong potential as a marker of urban aesthetics. In particular, the superior longitudinal fasciculus (SLF), the brain's largest associative tract (Janelle et al. 2022), interconnects frontal, temporal, and parietal structures through fibers passing through the inferior parietal, premotor, prefrontal, medial and inferior frontal, postcentral, and superior temporal regions (Makris et al. 2005). Given its involvement in higher order cognitive functions such as language processing, attention, working memory, somatosensory monitoring, and visuospatial perception (Bernal and Altman 2010; Hoeft et al. 2007; Karlsgodt et al. 2008), the SLF is expected to be associated with urban aesthetics, which integrates diverse elements. Because the SLF connects frontal, parietal, and temporal regions involved in perception, attention, and higher order cognitive integration, it may provide a structural pathway supporting complex perceptual judgments such as urban aesthetic evaluation.

Therefore, the present study aims to clarify the relationship between subjective urban aesthetics and brain structure. Brain structure was assessed using FA and GMV, measured by the FA brain healthcare quotient (FA‐BHQ) and gray matter (GM)‐BHQ, developed by Nemoto et al. (2017). These indices have recently been shown to be associated with aesthetic appreciation (Kokubun et al. 2025), local environment (Kokubun, Nemoto, and Yamakawa 2025), and residential environment (Pineda et al. 2021). In this study, we hypothesized and tested that whole‐brain FA and SLF FA would be correlated with urban aesthetics, particularly with subjective evaluations of urban aesthetic quality.

## 2 Research Methodology

### 2.1 Participants

A total of 82 adults (52 males and 30 females; age range = 22–65 years, mean = 39.27, standard deviation [SD] = 10.42) participated in this study. Participants were recruited between June and July 2025 at the Institute of Science Tokyo through the BHQ Consortium, an industry–academia initiative focused on the application of brain‐related data.

Detailed information on participants’ occupations and job titles is presented in Table 1. The sample consisted of 63.4% males and 36.6% females, indicating a higher proportion of men compared with national workforce statistics in Japan (54.5% male, 45.5% female; Ministry of Internal Affairs and Communications, 2024). In addition, 18.5% of participants held managerial positions, which exceeds the national estimate of 11.5% reported for 2021 (Selection and Variation 2024).

**TABLE 1:** Participant's gender, occupation, and position.

	Freq.	%
*Sex*		
Male	52	63.4
Female	30	36.6
*Occupations*		
Managerial	15	18.3
Research and technical	6	7.3
Professional in legal affairs, Management, culture and arts, etc.	14	17.1
Medical, nursing and health care	4	4.9
Childcare and education	3	3.7
Administrative	6	7.3
Sales and sales	9	11
Welfare and caregiving	1	1.2
Service	23	28
Construction, civil engineering, and electrical construction	1	1.2
*Job titles*		
Executives and executives	17	20.7
Department heads	6	7.3
Managers and leaders	20	24.4
General and professional	26	31.7
Contract and self‐employed	7	8.5
Other	6	7.3

The target sample size was determined a priori using G*Power (version 3.1.9.7), assuming a medium effect size (*r* = 0.30; Cohen 1988), an alpha level of 0.05, and statistical power of 0.80. All participants self‐reported no history of neurological, psychiatric, or other conditions affecting the central nervous system.

MRI scans were conducted prior to questionnaire completion. The study protocol was reviewed and approved by the Ethics Committee of the Institute of Science Tokyo (Approval No. 2023137). All procedures complied with institutional guidelines and the principles of the Declaration of Helsinki. Written informed consent was obtained from all participants, and anonymity was ensured throughout the study.

The present dataset was derived from the same participant cohort used in our previous study examining different behavioral correlates of brain structure, but the present study addresses a distinct research question focusing on urban aesthetic perception and WM microstructure (Kokubun et al. 2026).

### 2.2 Psychological Scale

We used the Japanese version (Ohga et al. 2010) of the neighborhood environment scale developed by Mujahid et al. (2007). This scale defines the neighborhood as within a one‐mile radius from the respondent's home and assesses individuals’ perceptions of their neighborhood across 31 items covering seven dimensions: aesthetic quality, walking environment, availability of healthy foods, safety, violence, social cohesion, and activities with neighbors. The subscales have been validated by the developers, showing good internal consistency in a US sample (Cronbach's *α* = 0.75), test–retest reliability (*r* = 0.83), and correlations with census‐based measures of neighborhood socioeconomic status. In the present study, we used the aesthetic quality subscale. Aesthetic quality consists of five items rated on a five‐point Likert scale (strongly agree, agree, neutral, disagree, strongly disagree), such as “In my neighborhood the buildings and homes are well‐maintained.” Scores were calculated as the arithmetic mean of the items, ranging from 1 to 5, with higher scores indicating a more positive evaluation of urban aesthetics. In Japan, higher aesthetic quality scores have been associated with areas that are more systematically planned and have greater green space preservation, whereas lower scores have been linked to areas with more semi‐industrial zones and waste incineration sites.

### 2.3 MRI Data Acquisition

Structural MRI data were acquired at the Institute of Science Tokyo using a 3.0‐T MAGNETOM Prisma scanner (Siemens, Munich, Germany) equipped with a 32‐channel head coil. High‐resolution T1‐weighted anatomical images were obtained with a three‐dimensional magnetization‐prepared rapid gradient‐echo sequence. The acquisition settings were as follows: repetition time (TR) = 1900 ms, echo time (TE) = 2.52 ms, inversion time (TI) = 900 ms, flip angle = 9°, matrix = 256 × 256, field of view (FOV) = 256 mm, and slice thickness = 1 mm. Diffusion‐weighted images were collected separately using a spin‐echo echo‐planar imaging sequence with generalized autocalibrated partially parallel acquisition (GRAPPA). DTI data were acquired using a diffusion‐weighted spin‐echo echo‐planar imaging sequence. The acquisition parameters were as follows: TR = 9000 ms, TE = 81 ms, flip angle = 90°, acquisition matrix = 114 × 114, voxel size = 2.0 × 2.0 × 2.0 mm^3^, slice thickness = 2 mm, and 30 non‐collinear diffusion gradient directions with *b* values of 0 and 1000 s/mm^2^.

### 2.4 MRI Data Analysis

T1‐weighted anatomical images were processed using Statistical Parametric Mapping 12 (SPM12; Wellcome Trust Centre for Neuroimaging, London, UK) implemented in MATLAB R2022b (MathWorks Inc., Sherborne, MA, USA). First, each image was segmented into GM, WM, and cerebrospinal fluid (CSF). The resulting GM images were then normalized to standard space using the diffeomorphic anatomical registration through exponentiated Lie algebra (DARTEL) procedure (Ashburner 2007), with modulation applied to preserve local volumetric information. Subsequently, the normalized GM images were smoothed with an 8‐mm full‐width at half‐maximum (FWHM) Gaussian kernel. To account for individual differences in head size, the smoothed GM images were adjusted by intracranial volume (ICV), yielding proportional GM maps that were then used to calculate mean and SD reference images. By averaging this information and the local GM quotients using the automatic anatomical labeling (AAL) atlas, the GM‐BHQ was created with a mean of 100 and an SD of 15 points.

DTI data were preprocessed using the FMRIB Software Library (FSL) version 5.0.11 (Jenkinson et al. 2012). First, all diffusion‐weighted images were aligned to the first b0 image, and eddy current and head motion corrections were applied. FA images were then calculated using DTIFit based on tensor fitting. Afterward, FA images were spatially normalized to the standard Montreal Neurological Institute (MNI) space using FLIRT followed by FNIRT. Individual FA quotient images were generated using the formula 100 + 15 × (individual FA − mean)/SD to create participant‐specific FA‐BHQ values using the Johns Hopkins University (JHU) DTI‐based WM atlas (Mori et al. 2008). This standardization follows the Brain Healthcare Quotient framework proposed by Nemoto et al. (2017), in which the mean is set to 100 and the SD to 15, analogous to an intelligence quotient, to facilitate intuitive interpretation and comparability across studies. For more details, see Nemoto et al. (2017) and Kokubun et al. (2023).

Some elements of the participant cohort and MRI preprocessing framework overlap with our previously published study using the same dataset but addressing a different behavioral domain (Kokubun et al. 2026).

## 3 Analysis

First, correlation coefficients were calculated between aesthetic quality, whole‐brain FA, SLF FA, and control variables. In calculating the correlation coefficient, the Shapiro–Wilk normality test confirmed that aesthetic quality did not follow a normal distribution, so Spearman's ρ was adopted. Subsequently, a hierarchical multiple regression analysis was conducted with whole‐brain FA and SLF FA as the dependent variables and aesthetic quality and control variables as the independent variables. For reference, an explorative additional analysis was performed in which the dependent variable was replaced with AAL 116 subregions of GMV and AAL 48 regions of FA.

Control variables included sex (male = 1, female = 0), age (years old), education (years), body mass index (BMI: kg/m^2^), systolic blood pressure (mmHg), diastolic blood pressure (mmHg), pulse (times/min), stress, sleep disorders, exercise, and happiness. These indicators have been confirmed to be related to GMV and FA (e.g., Kokubun, Nemoto, and Yamakawa 2025).

Stress levels were operationalized based on responses to the question, “Do you often feel stressed these days?” using a 4‐point scale: “rarely,” “sometimes (a few times per month),” “frequently (at least once a week),” and “almost always.” Each response was assigned a score from 1 to 4, which was used as the stress variable.

Sleep disorders were assessed by answering the question “How do you feel about the quality of your sleep these days?” using a four‐point scale: “I'm sleeping soundly,” “My sleep is a little shallow, but I'm getting enough sleep,” “I often have shallow sleep and feel tired,” and “I wake up many times during the night and can't get a good night's sleep,” and the responses were assigned a score of 1–4, creating a variable.

Physical activity (PA) was assessed using the Japanese version of the International PA Questionnaire—Short Form (IPAQ‐SF). Responses regarding average time per day and frequency per week for vigorous, moderate, and light exercise were quantified using the Metabolic Equivalent of Task (MET) following the IPAQ guideline (CRAIG et al. 2003).

Happiness was quantified by assigning a score of 1–7 to responses on a 7‐point scale ranging from “disagree” to “agree” to the statement “Overall, I am happier than people my age,” which is a slightly modified version of one of the four items that make up the subjective happiness scale developed by Lyubomirsky and Lepper (1999).

The significance level was set at 5%. Because two regression models were tested (whole‐brain FA and SLF FA), Bonferroni correction was applied to control for multiple comparisons, resulting in a corrected significance threshold of *p* < 0.025 (=0.05/2 tests). In subsequent exploratory analyses, a conservative criterion of *p* < 0.001 was adopted, and the results of a more lenient *p* < 0.01 were also shown for reference. Following Cohen (1988), an increment in the coefficient of determination of Δ*R* ^2^ = 0.02 or more and Δ*R* ^2^ = 0.13 or more were used as the criteria for effect sizes exceeding “small” and “moderate,” respectively. All statistical analyses were performed using IBM SPSS Statistics/AMOS Version 26 (IBM Corp., Armonk, NY, USA).

## 4 Results

**FIGURE 1:**
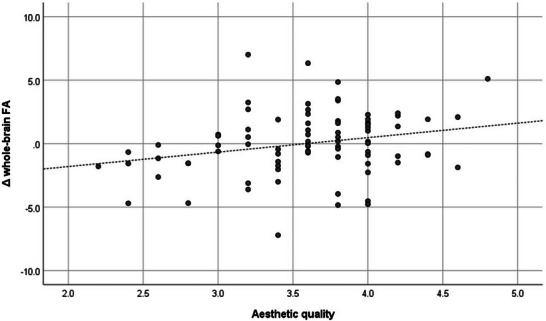
Scatter plot showing the correlation between aesthetic quality and whole‐brain FA (*r* = 0.248). FA, fractional anisotropy. The vertical axis shows residuals from a multiple regression model including only the control variables.

**FIGURE 2:**
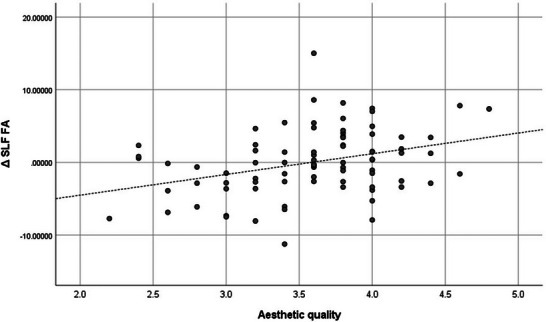
Scatter plot showing the correlation between aesthetic quality and SLF FA (*r* = 0.344). SLF FA, superior longitudinal fasciculus fractional anisotropy. The vertical axis shows residuals from a multiple regression model including only the control variables.

Table 2 shows the correlation coefficients between variables. Whole‐brain FA (*r* = 0.171, *p* = 0.125) did not show a significant positive correlation with aesthetic quality, but SLF FA (*r* = 0.297, *p* = 0.007) did. Table 3 shows the results of hierarchical multiple regression analysis. Control variables were added in Step 1, and aesthetic quality was added in Step 2. All figures in the table are for Step 2, except for the change in effect size Δ*R* ^2^ from Step 1 to Step 2 shown at the bottom. Both whole‐brain FA (β = 0.273, *p* = 0.021, Δ*R* ^2^ = 0.061) and SLF FA (β = 0.397, *p* = 0.001, Δ*R* ^2^ = 0.130) showed a significant positive correlation with aesthetic quality. The effect size for the former exceeded the “small” criterion of Δ*R* ^2^ = 0.02, whereas the latter was equal to the “moderate” criterion of Δ*R* ^2^ = 0.13. This result supports the hypotheses of this study. Figures 1 and 2 show the correlations between aesthetic quality and whole‐brain FA and SLF FA in scatter plots.

**TABLE 2:** Descriptive statistics and correlation analysis results.

		Mean	SD		1	2	3	4	5	6	7	8	9	10	11	12	13
1	Sex	0.6	0.5	*r*													
				*p*													
2	Age (years)	39.3	10.4	*r*	0.249^*^												
				*p*	0.024												
3	Education (years)	16.3	2.3	*r*	0.068	0.140											
				*p*	0.545	0.208											
4	BMI (kg/m^2^)	53.2	277.4	*r*	0.430^***^	0.354^**^	−0.004										
				*p*	<0.001	0.001	0.971										
5	Systolic BP (mmHg)	110.6	15.9	*r*	0.512^***^	0.348^**^	0.140	0.551^***^									
				*p*	<0.001	0.001	0.210	<0.001									
6	Diastolic BP (mmHg)	71.5	12.5	*r*	0.224^*^	0.438^***^	0.137	0.287^**^	0.728^***^								
				*p*	0.043	<0.001	0.220	0.009	<0.001								
7	Pulse (times/min)	75.4	11.8	*r*	0.007	0.185	0.021	0.132	0.104	0.307^**^							
				*p*	0.947	0.096	0.853	0.237	0.353	0.005							
8	Stress	2.2	0.8	*r*	−0.077	−0.221^*^	−0.065	−0.024	−0.025	0.037	0.036						
				*p*	0.493	0.046	0.563	0.831	0.823	0.738	0.751						
9	Sleep disorders	2.0	0.9	*r*	−0.099	−0.142	−0.056	0.095	−0.019	−0.031	−0.002	0.372^**^					
				*p*	0.378	0.203	0.616	0.396	0.864	0.780	0.983	0.001					
10	Exercise	1731.3	1867.0	*r*	0.064	−0.227^*^	−0.100	0.108	0.018	−0.140	−0.240^*^	−0.092	−0.056				
				*p*	0.570	0.040	0.371	0.334	0.875	0.211	0.030	0.413	0.615				
11	Happiness	5.2	1.5	*r*	0.092	0.121	−0.017	0.005	0.095	0.131	0.001	−0.400^***^	−0.457^***^	0.195			
				*p*	0.410	0.281	0.881	0.967	0.397	0.242	0.991	<0.001	<0.001	0.080			
12	Aesthetic quality	3.6	0.5	*r*	−0.032	0.139	0.017	0.042	0.070	0.055	−0.068	−0.191	−0.214	0.057	0.275^*^		
				*p*	0.773	0.213	0.879	0.71	0.534	0.623	0.542	0.086	0.053	0.613	0.012		
13	Whole‐brain FA	99.7	2.8	*r*	−0.120	−0.185	0.053	−0.019	−0.080	−0.157	−0.076	0.104	0.171	−0.022	−0.250^*^	0.171	
				*p*	0.284	0.096	0.638	0.866	0.474	0.158	0.498	0.352	0.125	0.845	0.023	0.125	
14	SLF FA	99.9	4.8	*r*	0.009	−0.047	0.104	0.021	0.034	−0.029	0.011	0.073	0.243^*^	−0.120	−0.088	0.297^**^	0.552^***^
				*p*	0.939	0.677	0.352	0.851	0.764	0.797	0.925	0.512	0.028	0.282	0.434	0.007	<0.001

*Note: N* = 82.

Abbreviations: BMI, body mass index; BP, blood pressure; FA, fractional anisotropy; SD, standard deviation; SLF FA, superior longitudinal fasciculus fractional anisotropy.

^*^ *p* < 0.05.

^**^ *p* < 0.01.

^***^ *p* < 0.001.

**TABLE 3:** Results of multiple regression analysis.

	Whole‐brain FA		SLF FA	
	*β*	*p*	*β*	*p*
Sex	0.011	0.930	0.126	0.332
Age (years)	−0.225	0.083	−0.047	0.716
Education (years)	0.047	0.668	0.110	0.320
BMI (kg/m^2^)	−0.152	0.183	−0.018	0.875
Systolic BP (mmHg)	0.046	0.823	0.008	0.968
Diastolic BP (mmHg)	−0.154	0.438	−0.198	0.323
Pulse (times/min)	0.090	0.429	0.106	0.357
Stress	−0.067	0.598	0.059	0.644
Sleep disorders	0.134	0.282	0.284	0.026^*^
Exercise	−0.001	0.995	−0.031	0.785
Happiness	−0.214	0.104	0.020	0.878
Aesthetic quality	0.273	0.021^*^	0.397	0.001^**^
*R* ^2^	0.243		0.231	
Δ*R* ^2^	0.061		0.130	

*Note: N* = 82.

Abbreviations: BMI, body mass index; BP, blood pressure; FA, fractional anisotropy; SLF FA, superior longitudinal fasciculus fractional anisotropy.

^*^ *p* < 0.05.

^**^ *p* < 0.01.

^***^ *p* < 0.001.

When we performed the same analysis as in Step 2 of Table 3 on the 116 GMV subscales and the 48 FA subscales, the correlation between aesthetic quality and right SRL (β = 0.402, *p* < 0.0008, Δ*R* ^2^ = 0.133) met the conservative criterion of *p* < 0.001 and was significant. The effect size also exceeded the “moderate” criterion of Δ*R* ^2^ = 0.13. Other significant effects, using the more lenient *p* < 0.01 criterion, were observed for the middle cerebellar peduncle FA (β = 0.368, *p* = 0.002, Δ*R* ^2^ = 0.111), splenium of corpus callosum FA (β = 0.349, *p* = 0.001, Δ*R* ^2^ = 0.101), right posterior corona radiata (β = 0.343, *p* = 0.004, Δ*R* ^2^ = 0.097), left SRL (β = 0.329, *p* = 0.008, Δ*R* ^2^ = 0.089), and right occipital superior (β = 0.309, *p* = 0.003, Δ*R* ^2^ = 0.078). All effect sizes exceeded the “small” criterion of Δ*R* ^2^ = 0.02.

## 5 Discussion

This study is the first to demonstrate an association between urban aesthetics and brain structure. Previous studies using fMRI have measured neural responses and revealed that a wide range of brain regions respond to urban aesthetics. Neural responses do not necessarily produce permanent changes, but in light of discussions on neuroplasticity, repeated exposure may lead to lasting alterations (Strang 2024). In other words, the present findings suggest that the beauty of cities may bring about enduring changes in the human brain to a greater extent than previously believed. Moreover, these results provide implications for recent discussions in neuro‐urbanism, which focus on addressing global urban challenges by examining how urban life influences stress levels and mental health (Elsayed et al. 2024). Urban aesthetic experiences may promote positive emotional states, attentional engagement, and cognitive exploration. Repeated exposure to aesthetically pleasing environments could strengthen frontoparietal connectivity involved in perception, evaluation, and spatial cognition. Because the SLF connects frontal and parietal regions supporting these processes, structural differences in this pathway may partly reflect cumulative environmental aesthetic experiences.

It is also worth noting that the present findings emerged for FA rather than GMV. Previous studies have shown that various brain regions interact in response to beauty, suggesting that GMV—whether examined in individual regions or as an averaged value—may not sufficiently capture individual differences in urban aesthetic perception. In contrast, FA reflects the quality of pathways between regions, making it a potentially more suitable measure for predicting associations with urban aesthetics, which involve multiple sensory modalities such as vision, hearing, and smell. In particular, considering that the SLF constitutes the largest WM tract connecting regions implicated in urban aesthetics—including the PFC (e.g., frontopolar and superior frontal gyri) involved in conscious reasoning and analysis (Vartanian et al. 2013), the parahippocampal region associated with spatial navigation (Mégevand et al. 2014), occipital regions involved in perceptual feature processing (Geisler 2007), and the core reward‐related circuitry, including the OFC, ACC, and anterior insula (Brown et al. 2011)—as well as broader networks underlying aesthetic experience spanning from occipital to frontal cortices (Boccia et al. 2016), the present findings are consistent with prior research. Recent studies have also suggested that the SLF plays an important role in metacognitive evaluation and subjective decision‐making processes (Zheng et al. 2021; Zheng et al. 2025). Because aesthetic judgments involve subjective evaluation and reflective processes, the present findings linking SLF microstructure with urban aesthetic perception are consistent with this emerging literature.

However, the present findings allow for alternative interpretations. For instance, in light of recent studies suggesting that urban green space exposure may promote WM integrity (Binter et al. 2024; Liu et al. 2025), it is possible that the association between urban aesthetics and FA is mediated by stress. Under such an interpretation, the relationship between beauty and the brain proposed by neuroaesthetics researchers would be indirect, with stress acting as the proximal factor exerting direct effects on FA. Nevertheless, because the present findings were obtained after controlling for subjective stress using a simplified measure, it is more likely that urban aesthetics independently contributes to FA, beyond the influence of subjective stress. It should also be noted that FA reflects multiple microstructural properties, including axonal density, myelination, and fiber organization. Because FA lacks biological specificity, the present findings should be interpreted cautiously, and future studies incorporating complementary diffusion metrics may help clarify the underlying neurobiological mechanisms.

Nevertheless, in light of a series of prior studies showing that exposure to air pollution (Cserbik et al. 2020; Guxens et al. 2018; Lubczyńska et al. 2021) and noise (Binter et al. 2024) may affect the microstructural connectivity between limbic and frontotemporal networks (Lautarescu et al. 2020), it is plausible that the relationship between urban aesthetics and FA involves complex, intertwined factors. For example, in less aesthetically pleasing urban environments, problems such as pollution and noise may be more severe, which could independently elevate the release of stress hormones—apart from subjective stress—and thereby influence FA (Hahad et al. 2019).

Alternatively, as suggested by a recent meta‐analysis showing that negative aesthetic evaluations, or the presentation and evaluation of negative non‐artistic images, are associated with activation not only in the right fusiform gyrus but also across frontal, occipital, temporal, and subcortical regions (Slaby et al. 2025), the present findings may reflect the extent of negative rather than positive aesthetic evaluations. In such a case, the policy implications could differ substantially: instead of prioritizing efforts to enhance urban beauty, it might be a more rational policy choice to focus on mitigating negative aspects. Future studies should employ more nuanced scales to test the questions that emerge from the present work.

The present findings, obtained from adults with self‐reported good health, suggest that the way cities are designed may influence human well‐being to a greater extent than previously assumed. Urban development may need to be guided not only by practical convenience but also by considerations of beauty, in ways that foster brain health. This discovery can serve as a first step toward revisiting and reconstructing the fundamental question of why people choose to live in cities.

## 6 Limitation

This study has several limitations. First, because of the cross‐sectional design, we cannot directly estimate the causal relationship between aesthetic quality, whole‐brain FA, and SLF FA. In addition, environmental exposures during early development, including prenatal noise or pollution exposure, may influence WM development. Because the present study focused on adults, the observed associations cannot exclude the influence of earlier developmental factors. Second, the measurement of aesthetic quality was based on self‐reporting and is not an objective behavioral indicator. Third, the small sample size reduces the generalizability of the results. Fourth, because the study was conducted on Japanese subjects, caution should be exercised when applying the results to people in other countries. Fifth, the participants were biased towards men and managers, so there may be a discrepancy with the average Japanese trend. Sixth, the aesthetic quality scale used in this study consisted of only five items each; therefore, reproducibility should be tested using a more comprehensive scale. In particular, by taking into account the type of beauty (vision, hearing, smell, etc.) and the emotional experiences associated with beauty, it may have been possible to clarify the relationship with FA more precisely. Seventh, this study predicted the involvement of WM tracts connecting nerve cells based on previous research on urban beauty and other aesthetic experiences, but the possibility of other factors, such as stress reduction through exposure to green spaces or the effects of pollution and noise, cannot be ruled out. Therefore, unmeasured confounding factors not included in the analysis model may have influenced the results, potentially biasing the observed associations. Finally, because this study only used FA and GMV as an index of brain structure, future studies may consider incorporating other indices, such as intrinsic brain activity and connectivity (Ma et al. 2024) and structural‐functional connectivity (Suo et al. 2025), to gain a more comprehensive understanding of the neurobiological mechanisms associated with urban aesthetic perception. Future research should verify the results of this study through cross‐sectional and longitudinal studies that include a large number of participants of diverse nationalities in a more comprehensive way. Future studies should also compare urban aesthetic perception with other forms of aesthetic experience, such as artistic, architectural, and natural landscape aesthetics, to clarify the domain specificity of the present findings.

## 7 Conclusion

The growing interest in the relationship between urban aesthetics and the brain is reflected in emerging movements such as neuro‐urbanism. However, the association between urban aesthetics and brain structure has remained unclear. Using DTI, the present study demonstrated positive associations between subjective urban aesthetics and both whole‐brain FA and SLF FA.

## Author Contributions

**Keisuke Kokubun**: conceptualization, formal analysis, writing – original draft. **Kiyotaka Nemoto**: methodology, data curation, funding acquisition, investigation, administration, supervision, writing – review and editing. **Maya Okamoto**: methodology, data curation, funding acquisition, investigation, administration, supervision, writing – review and editing. **Yoshinori Yamakawa**: methodology, data curation, funding acquisition, investigation, administration, supervision, writing – review and editing. All authors reviewed the final manuscript.

## Funding

This work was funded by the ImPACT Program of the Council for Science, Technology and Innovation (Cabinet Office, Government of Japan) and supported by JSPS KAKENHI (Grants JP17H06151 and JP25K15384).

## Ethics Statement

This study was approved by the Ethics Committee of Institute of Science Tokyo (Approval Number 2023137) and was conducted following the institute's guidelines and regulations. All participants provided written informed consent before participation, and their anonymity was maintained.

## Consent

All participants gave consent for the publication of the results of this study.

## Conflicts of Interest

The authors declare no conflicts of interest.

## Data Availability

The datasets generated during the current study are not publicly available but are available from the corresponding author upon reasonable request.
